# Management of Peptide Receptor Radionuclide Therapy Toxicities in Neuroendocrine Neoplasm Patients

**DOI:** 10.1007/s11864-026-01379-z

**Published:** 2026-01-16

**Authors:** Chirayu Mohindroo, Robert A. Ramirez

**Affiliations:** 1 National Cancer Institute, National Institutes of Health, Bethesda, MD USA; 2 Vanderbilt University Medical Center, Division of Hematology and Oncology, 2220 Pierce Ave., 7770 Preston Research Building, Nashville, TN 37232 USA

**Keywords:** Peptide receptor radionuclide therapy, Gastroenteropancreatic neuroendocrine neoplasms, Lutetium-177 DOTATATE, Treatment toxicity, Multidisciplinary care

## Abstract

Peptide receptor radionuclide therapy (PRRT) has become an established treatment for patients with well-differentiated gastroenteropancreatic neuroendocrine neoplasms (GEP-NENs) that express somatostatin receptors. Among the available agents, lutetium-177 DOTATATE is the most commonly used radiolabeled somatostatin analog and has demonstrated significant clinical benefits, including improved response rates and prolonged progression-free survival, as shown in landmark trials such as NETTER-1 and NETTER-2. As the incidence of GEP-NENs continues to rise, the use of PRRT in clinical practice has grown accordingly. However, this therapy is associated with a range of toxicities that can affect multiple organ systems over different timeframes, including acute, subacute, and long-term periods. This review provides a comprehensive overview of the adverse effects associated with PRRT and presents evidence-based strategies for their monitoring, prevention, and management. This review also discusses the evolving paradigm of screening for clonal hematopoiesis before PRRT treatment, as well as the use of steroids as prophylaxis to prevent carcinoid crisis and bowel obstruction. A multidisciplinary approach is essential to ensure the safe delivery of treatment, early detection of complications, and tailored patient care. As clinical experience with PRRT expands, continued refinement of supportive care strategies will be critical to optimizing outcomes and minimizing toxicity in this complex patient population.

## Introduction

**Fig. 1:**
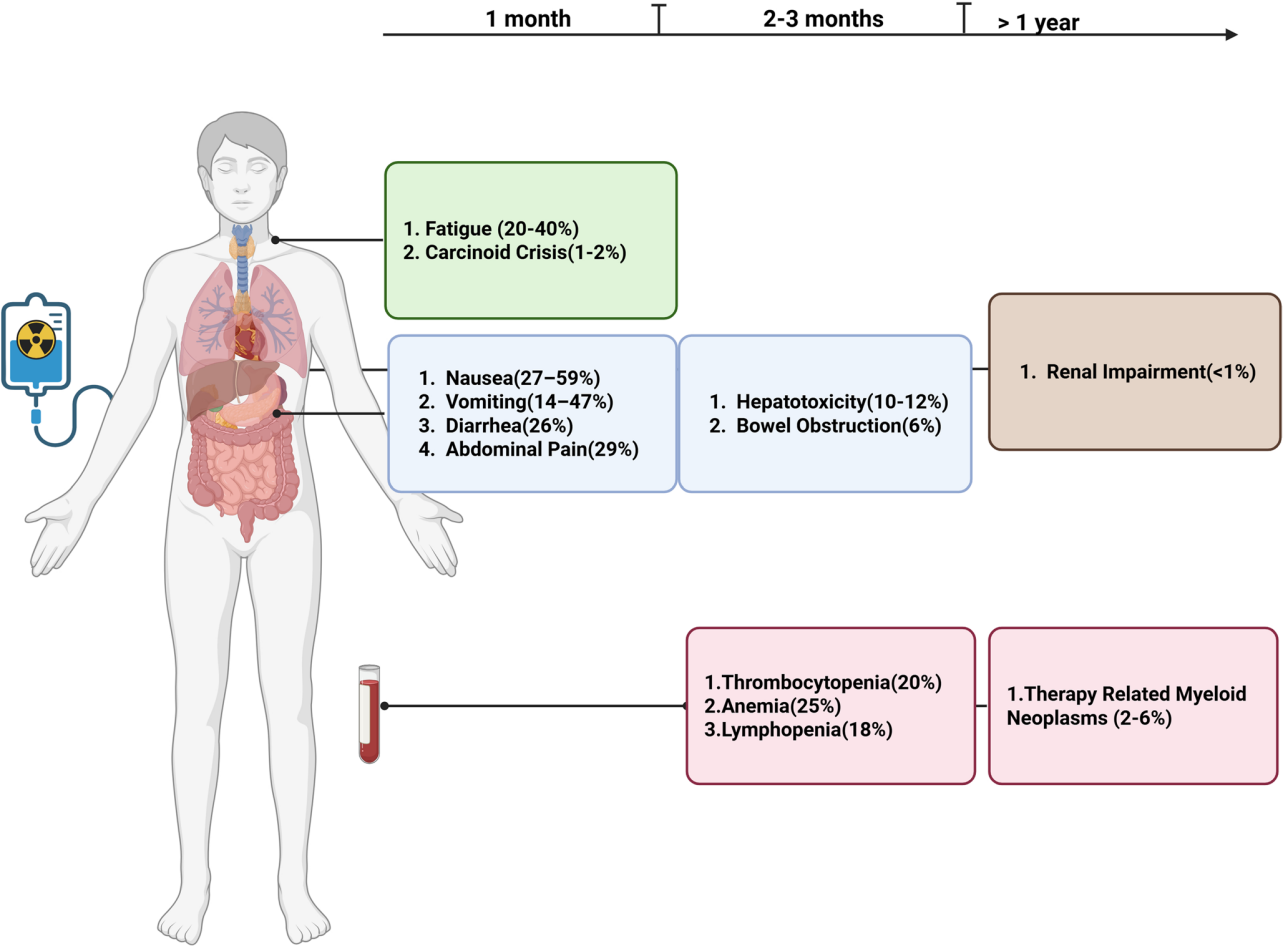
Schematic illustration of a patient receiving Peptide Receptor Radionuclide Therapy (PRRT), with organ-specific toxicities shown along a timeline. Colored boxes indicate the affected organ systems: blue boxes represent gastrointestinal toxicities, red boxes represent hematologic toxicities, brown boxes indicate renal toxicity, and green boxes represent systemic symptoms. The boxes are positioned anatomically along the patient’s body and temporally from acute to long-term, illustrating the distribution and progression of PRRT-associated toxicities. Prevalence of each adverse effect is noted in the brackets. Created in BioRender. Mohindroo, C. (2025) https://BioRender.com/alf86oh

The role of peptide receptor radionuclide therapy (PRRT) is now well established for well-differentiated gastroenteropancreatic neuroendocrine neoplasms (GEP-NEN). GEP-NENs expressing somatostatin receptors (SSTR) can be targeted with radiolabeled somatostatin analogs (SSAs) using alpha- and beta-emitting radionuclides. As the incidence and prevalence of GEP-NETs has been rapidly increasing over the past two decades [1], the use of PRRT has also consequently increased. While alpha-emitting radionuclides are being explored, beta emitters remain the mainstay of clinical practice. The most commonly used radiopharmaceutical being is ^177^Lu-DOTATATE approved for well differentiated advanced somatostatin receptor positive GEP-NENs [2]. The United States Food and Drug Administration approval in 2018 was based on the NETTER-1 trial showing increased progression free survival and response rate compared to high dose octreotide only with a manageable safety profile [2]. The follow up study, NETTER-2, showed that for select patients PRRT might also be appropriate in the first line setting [3]. As longer follow-up from prospective clinical trials [4, 5] and more real-world data [6–8] have emerged, our understanding of the safety profile of PRRT has continued to improve. While the treatment targets tumor cells, the beta radiation also damages DNA and cellular structures in surrounding tissues and can be absorbed by other organs over time, potentially leading to acute, subacute, and long-term toxicities affecting various organ systems [9]. This review will focus on synthesizing the current literature on both common and rare toxicities associated with PRRT treatment **(**Fig. 1**)**, along with practical strategies for their management and prevention **(**Table 1**)** within a multidisciplinary care framework.

**Table 1:** Summary of PRRT-Related Toxicities, Incidence, and management strategies

Toxicity	Incidence	Management
Acute		
Nausea	27–59%	• **Pre-medication**: Anti-emetics (5-HT3 ± H2/NK-1 antagonists, steroids, benzodiazepines) • **Infusion adjustment**: Slow, gradual amino acid increase • **Dilution strategy**: Use more dilute amino acid preparations
Carcinoid crisis	1–2%	• **Primary therapy**: Administer SSA agents (e.g., octreotide bolus or drip) and vasopressors as needed. • **Refractory options**: Consider vasopressors, cyproheptadine, or telotristat • **Preventive measures**: Reduce tumor volume, use steroids ± 5-HT3 antagonists, avoid octreotide pre-PRRT, and ensure amino acid infusion with lysine/arginine
Subacute		
Hematologic toxicity	Anemia:14–20% Thrombocytopenia: 17–25% Lymphopenia:10–18%	• **Monitoring**: Baseline and follow-up CBCs every 2–4 weeks • **Supportive care**: Hold treatment, give transfusions or antibiotics as needed • **Dose adjustment**: Reduce dose in future cycles for thrombocytopenia
Hepatic Dysfunction	10–12%	• **Monitoring**: Check liver enzymes, bilirubin, and albumin at baseline and each cycle • **Intervention**: Stop hepatotoxic agents; hold PRRT for severe hepatotoxicity, then resume with dose reduction
Bowel Obstruction	6%	• **Primary approach**: Standard surgical management • **Risk stratification**: Identify high- and intermediate-risk patients based on tumor burden and bowel involvement • **Preventive/adjunct therapy**: Use prophylactic or therapeutic corticosteroids to reduce obstruction risk and inflammation
Long-Term Toxicities		
Therapy related myeloid neoplasms	2–6%	• **Management**: Refer to malignant hematology for t-MN treatment • **Prevention**: Screen for CH or cytopenias to detect high-risk patients, Apply risk-adapted methods to minimize radiation and optimize treatment sequencing
Renal Toxicity	1–2%	• Co-administer amino acids (arginine, lysine) to reduce renal radiation uptake

## Classification of PRRT-Related Toxicities

### Acute Toxicities

Acute toxicities are generally seen within 48 h of administration and up to a week. These generally include gastrointestinal side effects, hormonal crisis, and local infusion reactions.

1. **Nausea/vomiting**- Nausea and vomiting are among the most common adverse reactions associated with PRRT infusion. Large clinical trials have reported that 27–59% of patients experience nausea of any grade, while 14–47% experience vomiting [2, 3]. Similar findings have been observed in real-world data, with nausea reported in up to 48% of patients and vomiting in as many as 38%[10]. In addition to direct gastrointestinal irritation and, less commonly, hormone-related effects, the most frequent cause of nausea and vomiting during PRRT is the coadministration of amino acid infusions, which induce hyperosmolarity and subsequently lead to these symptoms [11].
  **Management**: Prior to administration a combination of anti-emetics such as 5-HT3 antagonist and in more severe cases H2 receptor antagonist or NK-1 receptor antagonist with steroids or benzodiazepines can be given. Additionally, slower infusion with a more gradual increase of the amino acids and instead of using commercially available preparations of amino acids preparing more a dilute version can also reduce the incidence of nausea/vomiting in severe cases [6, 11, 12].
2. **Carcinoid crises**: PRRT treatment can induce carcinoid crisis with the incidence being around 1–2%[13–16]. The most common presentation is within 12–48 h with the first dose, and symptoms include hypotension, nausea/vomiting, severe diarrhea, tachycardia, altered mental status and flushing [17]. Rarely, cardiovascular collapse can also be seen [18]. Major risk factors include high levels of serum or urine 5-hydroxyindolacetic acid, higher tumor burden, recent discontinuation of SSA agents, metastatic disease to the liver, and old age [5, 14, 16, 19]. The potential mechanism of carcinoid crisis remains controversial: it was previously thought to represent tumor lysis with massive hormone surge, but recent evidence suggests that high baseline hormone levels may predict risk and that hormone levels can actually fall during the crisis [18, 20, 21].
  **Management**: Ensuring airway and breathing is of paramount importance. Traditionally, administration of a SSA agents such as Octreotide can be given as intravenous boluses of 500–1000 ug till symptom control, and a drip can also be considered in refractory cases [16]. Recent evidence suggests that the mechanism of action of octreotide during carcinoid crisis may be mediated through splanchnic vasoconstriction rather than reversal of hormone effects [18, 20, 21], and studies also indicate that vasopressors may be more effective than octreotide in treating these events [22]. Other reported options in refractory cases include using cyproheptadine (5-HT2A receptor inhibitor), and telotristat ethyl (Tryptophan hydroxylase inhibitor) [13, 16, 23, 24]. Various techniques to reduce carcinoid crisis have been proposed. In patients with high tumor volume, reducing the tumor volume via surgery, radioembolization or radiation treatment can reduce carcinoid crisis [19]. In terms of pharmacological management, giving steroids alone or in combination with selective 5-hydroxytryptamine 3 receptor antagonists and intravenous fluids can be considered [5, 13, 14, 19, 25]. Notably, long and short acting octreotide should be avoided 24 h prior to PRRT as studies have reported that it may impact efficacy by direct competitive inhibition for uptake into tumor cells [16, 26]. Additionally, avoiding exercise and administering an amino acid infusion high in lysine and arginine have both been shown to reduce the occurrence of carcinoid crisis [16, 19].
3. **Asthenia/fatigue**: Asthenia or fatigue is one of the most common symptoms associated with PRRT treatment. Prevalence in clinical trials with PRRT group ranges from 20–40%[2, 3] and similarly in real world studies(50%)[27]. The onset is generally within hours post infusion and can last for days. The cause of fatigue is generally multifactorial secondary to myelosuppression, systemic inflammatory response, poor oral intake from gastrointestinal toxicities and other organ dysfunction such as kidney and liver. Pain and psychosocial factors may also play a role [28]. The symptoms tend to be mild-moderate but can reach grade 3 severity resulting in discontinuation of the treatment [2–4, 29].
  **Management**: There is growing recognition of fatigue in patients with GEP-NENs, and various quality-of-life assessment tools [28] are being used in research, though they have not yet been widely adopted in clinical practice. First-line management includes structured exercise programs, cognitive behavioral therapy, and mindfulness-based interventions [30]. Pharmacological management may be considered in rare cases, but only after a thorough evaluation for reversible causes, including organ dysfunction [30].
4. **Radiation Contamination**: Radiation contamination is a critical consideration in the administration of PRRT [31, 32]. Lutetium-177 emits both beta and gamma radiation during decay, posing a risk of external contamination to healthcare personnel during infusion and to close contacts of the patient following treatment. To minimize these risks, PRRT must be conducted in specialized nuclear medicine facilities with established radiation safety protocols [33]. All staff involved should be trained in radiation protection procedures, and patients are generally required to observe post-treatment isolation to reduce exposure to others [32]. Strict hygiene measures are recommended, including the use of a separate toilet, double flushing, and sleeping in a separate bed. These precautions are particularly important for incontinent patients, who may require catheterization to manage the risk of radioactive excreta. Additionally, the use of electronic medical record alerts and patient wristbands is advised to ensure healthcare providers are aware of recent PRRT administration [34].

Other acute toxicities including abdominal pain [2–4], infusion site reactions [35], tumor lysis syndromes [36], metabolic abnormalities [37] and hypersensitivity reactions have also been reported with PRRT [31]. Management generally follows standard oncology principles, tailored to the severity and underlying cause of each adverse event.

### Subacute/Delayed Toxicities

In addition to acute side effects, PRRT can lead to subacute toxicities that typically arise within 4–6 weeks following treatment. These toxicities may involve hematologic, hepatic, and gastrointestinal systems, and can vary in severity depending on patient-specific risk factors. Timely recognition and appropriate management are essential to minimize complications and maintain treatment continuity.

1. **Hematologic toxicity**: Radiation from PRRT can cause direct damage to the bone marrow, which can result in anemia(14–20%), thrombocytopenia(17–25%), lymphopenia(10–18%) and rarely pancytopenia(1%)[2, 3, 38]. Typically, the toxicities tend to be mild and reversible and seen within 4–6 weeks of administration of PRRT [39]. Approximately 0.1 and 0.4 Gy per infusion of 7.4 GBq can be absorbed by the bone marrow [38–41] and studies have reported dose-dependent relationship [39]. Large clinical trials have reported 5–10% of grade 3/4 hematological adverse events [2, 3]. Various risk factors have been associated with higher grade toxicities namely, kidney dysfunction, extensive bony disease, advanced age, prior history of chemotherapy and radiation treatment and preexisting cytopenias [38, 39, 42, 43]. Hematological toxicities have also limited combinations of PRRT with other agents such as chemotherapy [44].
  **Management**: Baseline complete blood counts (CBCs) should be performed in all patients, with repeat testing at 2–4 weeks and continued until any abnormalities are resolved [33]. Supportive management may include interruption of treatment while awaiting count recovery, occasional blood transfusions, and the use of antibiotics if needed. Dose reductions are generally considered for subsequent PRRT cycles, most commonly in cases of thrombocytopenia [38].
2. **Hepatic Dysfunction**: Direct radiation damage from PRRT can be associated with mild [2, 4, 6, 45] to severe [46] hepatotoxicity with an incidence of about 10–12%. Large clinical trials have not reported any grade 3/4 events [2, 4]. Patients with extensive (> 50%) hepatic metastasis [2, 47], preexisting liver dysfunction [48] and history of liver directed treatments may be at higher risk [49]. The manifestation is generally an increase in aminotransferases and hypoalbuminemia. Rare cases of liver failure have been reported but it is unclear if that is related to the disease biology or the PRRT itself [50, 51].
  **Hepatic Dysfunction**: Frequent monitoring of the liver enzymes, bilirubin and albumin is recommended at baseline and with each cycle [52]. Discontinuation of hepatotoxic agents is also recommended depending on the grade and severity of impairment. For significant hepatotoxicity (such as bilirubin > 3x ULN, Albumin < 30 g/L with INR > 1.5) holding the PRRT until resolution followed by dose reduction is recommended [52]. Exploratory reports have indicated that sequential chemotherapy before PRRT might improve response and reduce toxicity; however, this strategy remains investigational and is not part of standard practice [53].
  **Bowel Obstruction**: Patients with mesenteric or peritoneal involvement from small bowel NENs are at chronic risk of developing bowel obstruction. This is often due to desmoplastic fibrosis surrounding mesenteric metastases, which can cause retraction and fixation of adjacent bowel loops [54, 55]. Radiation delivered to these areas during PRRT may further contribute to obstruction risk by triggering local inflammation and exacerbating existing fibrotic changes [54, 55]. The prevalence is about 6% and seen generally within 3 months [56, 57]. Rarely, these toxicities can also be life-threatening [58, 59].
  **Management**: Standard surgical approaches remain the mainstay of treatment for bowel obstruction. However, there are currently no established guidelines for identifying high-risk patients or for preventing obstruction in the context of PRRT. Some studies suggest stratifying patients into high-risk and intermediate-risk groups. High-risk patients are characterized by extensive peritoneal implants, mesenteric masses, or omental caking, with significant bowel tethering that results in intermittent partial bowel obstruction. In contrast, intermediate-risk patients are defined as having scattered peritoneal or mesenteric metastases, a mesenteric mass with minimal bowel tethering, or implants in the rectovesical or rectouterine pouch. Management strategies differ between groups, with high-risk patients often directed toward alternative treatments, while intermediate-risk patients may receive prophylactic steroids. One proposed regimen includes 8 mg of intravenous dexamethasone after completion of the amino acid infusion, followed by a three-week oral taper beginning with 4 mg daily. Ongoing studies are evaluating whether alternative steroid regimens may further reduce the risk of obstruction [60]. In patients who develop bowel obstruction shortly after PRRT, corticosteroids may also be considered as part of the management strategy to reduce inflammation [56, 58].

In addition to the above, subacute toxicities such as hair loss [61], sexual dysfunction [62], and volume overload [63] may occur in certain patient populations, warranting careful monitoring and individualized management.

### Long-Term Toxicities

Chronic complications of PRRT primarily involve kidney damage and blood disorders such as leukemia or MDS, as the kidneys and bone marrow are the most sensitive organs to radiation exposure. These adverse effects generally emerge well beyond one year after the conclusion of PRRT therapy.

1. **Therapy related myeloid neoplasms**: The risk of developing long-term hematologic complications such as therapy-related myeloid neoplasms (tMN), including acute myeloid leukemia (AML) and MDS, following PRRT is not well-defined. Evidence available from both prospective trials and retrospective studies is confounded by prior exposure to alkylating agents such as temozolomide [64, 65] and previous radiation treatment, making it challenging to isolate PRRT as the primary causative factor. Large clinical trials with long-term follow-up (68–72 months) have reported that 2–6% of cases develop tMN [2, 4, 66]. Real-world studies [67, 68] have reported a higher prevalence (8–11%). Finally, meta-analyses have shown a risk of approximately 2.6% (range: 0–20%)[69]. Multiple mechanisms have been implicated in hematologic toxicity following PRRT [64], including prior exposure to alkylating agents, radiopharmaceutical absorption, and underlying genetic predispositions [64]. Clonal hematopoiesis (CH), particularly involving DNA damage response genes such as *TP53* and *PPM1D* [70], may provide mutant hematopoietic clones with a survival advantage under the selective pressure of treatments like PRRT. In addition, germline mutations in DNA repair genes [71, 72], identified in up to one third of patients with neuroendocrine tumors, may further increase susceptibility to t-MN[73], highlighting the importance of individualized risk assessment and prospective evaluation of dosing strategies.
  **Management**: Management of t-MNs following PRRT remains challenging, particularly in transplant-ineligible patients [74, 75], where treatment options are limited and outcomes are poor. Early identification of high-risk individuals through screening for CH[76] or cytopenias [77] may help guide treatment decisions and avoid PRRT in patients with elevated risk. Risk-adapted strategies that minimize cumulative radiation exposure and optimize treatment sequencing are critical to reducing the incidence of these aggressive hematologic complications [64]. Notably, retrospective evidence suggests that retreatment with PRRT does not increase the risk of t-MNs [69, 78]. This is currently being evaluated prospectively in the NET RETREAT trial (NCT05773274).
2. **Renal Toxicity**: The kidneys are considered dose-limiting organs in PRRT treatment due to their susceptibility to toxicity. The primary cause of renal irradiation is the filtration of radiolabeled peptides through the glomeruli, followed by their efficient reabsorption in proximal tubular cells, where the radioactive material can accumulate and remain for extended periods [79, 80]. Additionally, the presence of somatostatin receptor subtype in renal structures (cortical tubular cells, vasa recta and distal tubules) further enhances the renal uptake of radiolabeled somatostatin analogs [81]. The kidney dose limit for PRRT is estimated at 23 Gy from external beam data [82], but differences in radiation require long-term monitoring of renal toxicity, especially after multiple treatments. With modern techniques of renal protection with co-infusion of amino acids the incidence and severity have significantly decreased. NETTER-1 reported no nephrotoxicity [2, 4] and NETTER-2 reported 2% severe events in the PRRT arm vs. 1% in the octreotide arm [3]. Similarly real-world data is reassuring showing 0.7% grade 3/4 events with PRRT [83].
  **Management**: In addition to co-administering amino acids such as arginine and lysine to competitively reduce radiation uptake, caution is warranted when administering PRRT to patients with impaired renal function. Case reports have documented successful treatment in patients with a single kidney [84] as well as those on dialysis [85]. More recently, para-amino Hippurate has been explored as an alternative to amino acids due to its shorter administration time and fewer side effects [86]. In susceptible patient populations, dosimetry-guided PRRT may also be considered to optimize safety [85].

## Clinical Assessment and Laboratory Monitoring for PRRT

### Patient History

Detailed history should be gathered from the patient focusing on prior treatment received (especially liver-directed therapies, and somatostatin analogs), comorbidities (renal, hepatic, hematologic, depression and cardiovascular disease), symptoms of hormone hypersecretion (e.g., carcinoid syndrome, hypoglycemia) and performance status. Assessment of prior allergic reactions, especially to contrast agents or amino acids, and a detailed medication review are also essential [87, 88]. Genetic counseling and testing should be considered for patients with duodenal or pancreatic NENs or a family history suggestive of hereditary syndromes [71, 72, 89].

### Laboratory Tests

Baseline laboratory evaluations should include a complete blood count (CBC) with differential, renal function tests (serum creatinine and estimated glomerular filtration rate), liver function tests (AST, ALT, total bilirubin, and albumin), and electrolyte levels. These assessments are essential to determine patient eligibility and to identify those at higher risk for PRRT-related complications [33, 90]. For patients with symptoms suggestive of a functional tumor, relevant hormone assays (e.g., chromogranin A, 5-HIAA, insulin, gastrin, glucagon, VIP) should be performed [91]. Post-PRRT laboratory surveillance should be structured to detect emerging toxicities early. It is recommended to assess a CBC and comprehensive metabolic panel (CMP) at approximately 1, 3, 6, and 12 months after treatment. If values remain stable and within normal limits, annual monitoring of CBC and serum creatinine may be sufficient, unless earlier testing is warranted based on clinical symptoms or the judgment of the primary care or oncology team. In cases where abnormalities are detected, testing frequency should be increased to ensure timely intervention [11].

### Tumor Specific Testing

Tumor-specific evaluation should include histopathological confirmation with tumor grading based on the Ki-67 index, along with assessment of somatostatin receptor (SSTR) expression using SSTR PET/CT, such as [68]Ga-DOTATATE or [64]Cu-DOTATATE PET/CT, which is essential for determining eligibility for PRRT [33, 90]. Anatomic imaging with multiphasic contrast-enhanced CT or MRI of the abdomen and pelvis is recommended to evaluate tumor burden, hepatic involvement, and the presence of extrahepatic disease [33]. Both the National Comprehensive Cancer Network and the American Society of Clinical Oncology endorse these imaging modalities for staging and treatment planning. In cases of high-grade or poorly differentiated tumors, FDG PET/CT may also be indicated to assess tumor biology and guide therapeutic decisions [91]. Cardiac evaluation is also recommended if carcinoid heart disease is suspected [91].

## Multidisciplinary Management

Effective delivery of PRRT requires highly coordinated, multidisciplinary care involving NEN specialists including medical oncology, endocrinology, radiology, nuclear medicine or radiation oncology physicians, and care coordinators [92, 93]. Given the treatment’s complexity and infrastructure needs, many community practices refer patients to tertiary or academic centers with PRRT capabilities. Care coordinators play a central role in managing logistics, synchronizing workflows, and serving as liaisons between specialty teams and primary oncologists [88]. Early referral and collaboration are critical to ensure proper patient selection, streamline treatment, and ultimately return care closer to home whenever feasible. A clearly defined care pathway enhances patient outcomes and supports ongoing communication between all stakeholders [88]. Social work, physical therapy, and occupational therapy are essential components of multidisciplinary care for patients undergoing PRRT for neuroendocrine tumors. Social workers address psychosocial needs and care barriers, while physical and occupational therapists support mobility, energy conservation, and daily function. These allied health professionals collaborate closely with oncology and nuclear medicine teams to ensure safe treatment, enhance recovery, and maintain quality of life. This multidisciplinary approach is considered best practice in centers managing PRRT [87, 88].

## Conclusion

In conclusion, PRRT has become an established and effective treatment option for patients with well-differentiated GEP-NENs expressing somatostatin receptors. While it offers meaningful improvements in disease control and quality of life, PRRT is associated with a range of toxicities that can affect multiple organ systems and vary in timing and severity. Ongoing clinical trials, real-world data, and advancements in supportive care have enhanced our understanding of these adverse effects and informed practical strategies for prevention and management. A multidisciplinary approach remains essential to ensure safe delivery, timely recognition of complications, and individualized treatment planning.

## Key References

- Singh S, Halperin D, Myrehaug S, Herrmann K, Pavel M, Kunz PL, Chasen B, Tafuto S, Lastoria S, Capdevila J, García-Burillo A, Oh DY, Yoo C, Halfdanarson TR, Falk S, Folitar I, Zhang Y, Aimone P, de Herder WW, Ferone D; all the NETTER-2 Trial Investigators. [177Lu]Lu-DOTA-TATE plus long-acting octreotide versus high‑dose long-acting octreotide for the treatment of newly diagnosed, advanced grade 2–3, well-differentiated, gastroenteropancreatic neuroendocrine tumours (NETTER-2): an open-label, randomised, phase 3 study. Lancet. 2024 Jun 29;403(10446):2807–2817. 10.1016/S0140-6736(24)00701-3. Epub 2024 Jun 5.
  - ○ This reference is particularly important because it was the first large, randomized trial in treatment-naïve patients with neuroendocrine tumors. It provided key safety data and characterized the side-effect profile of PRRT compared with octreotide.
- Del Rivero J, Perez K, Kennedy EB, Mittra ES, Vijayvergia N, Arshad J, Basu S, Chauhan A, Dasari AN, Bellizzi AM, Gangi A, Grady E, Howe JR, Ivanidze J, Lewis M, Mailman J, Raj N, Soares HP, Soulen MC, White SB, Chan JA, Kunz PL, Singh S, Halfdanarson TR, Strosberg JR, Bergsland EK. Systemic Therapy for Tumor Control in Metastatic Well-Differentiated Gastroenteropancreatic Neuroendocrine Tumors: ASCO Guideline. J Clin Oncol. 2023 Nov 10;41(32):5049–5067. 10.1200/JCO.23.01529. Epub 2023 Sep 29. PMID: 37774329.
  - ○ This reference is of outstanding importance as it provides an evidence-based review of PRRT indications within the broader context of systemic therapy for metastatic GEP-NETs, identifies key gaps in long-term safety data, and reinforces the need for multidisciplinary collaboration in treatment planning and follow-up.

## Data Availability

No datasets were generated or analysed during the current study.
